# Supplementary material for: A CRISPR/Cas9 genetically engineered organoid biobank reveals essential host factors for coronaviruses
Source: Nat Commun. 2021 Sep 17;12:5498. doi: 10.1038/s41467-021-25729-7 (PMC8448725; doi:10.1038/s41467-021-25729-7)
Supplement: Supplementary file 1 — Supplementary Information [file 41467_2021_25729_MOESM1_ESM.pdf]

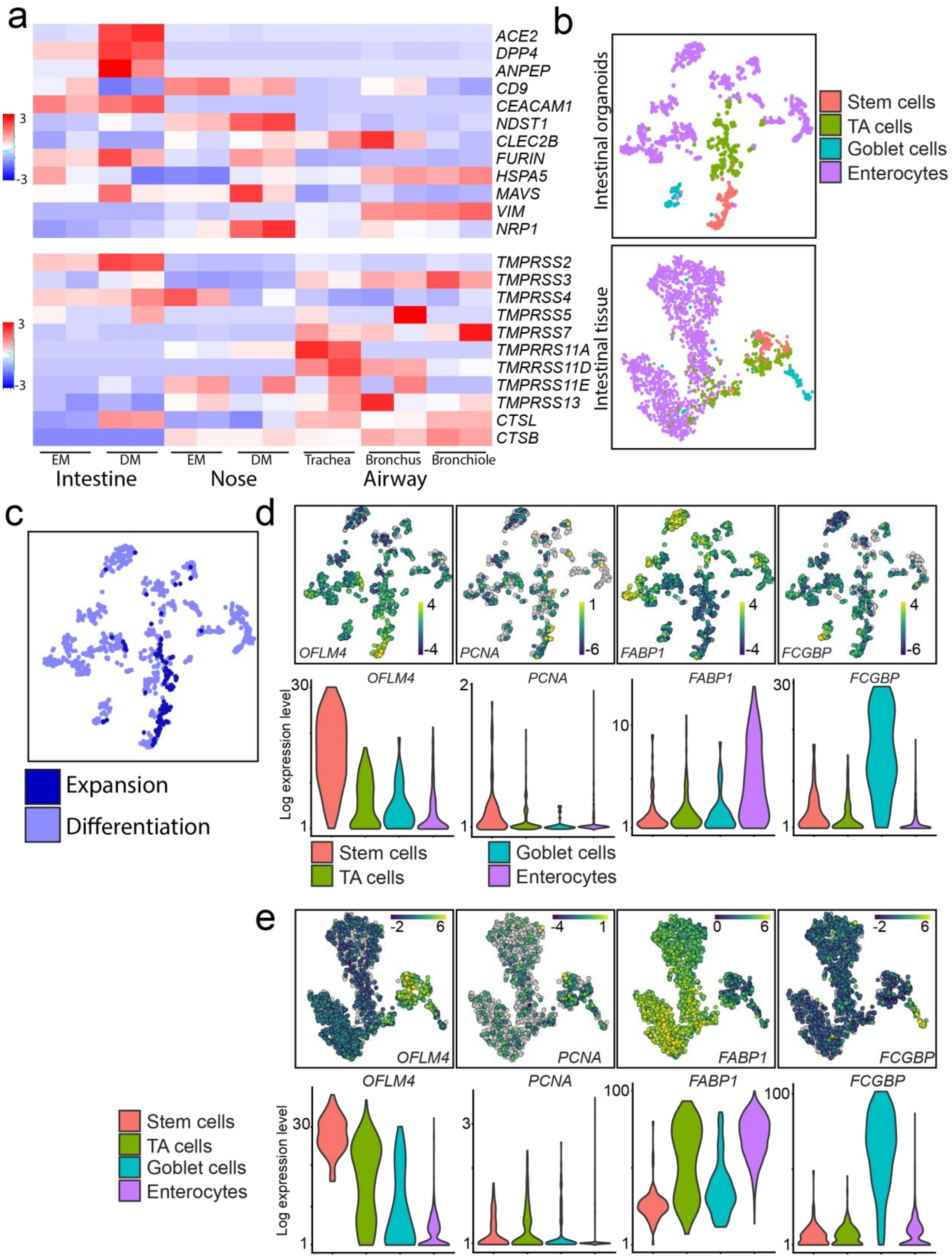

a
ACE2
DPP4
ANPEP
CD9
CEACAM1
NDST1
CLEC2B
FURIN
HSPA5
MAVS
VIM
NRP1
TMPRSS2
TMPRSS3
TMPRSS4
TMPRSS5
TMPRSS7
TMPRRS11A
TMRRSS11D
TMPRSS11E
TMPRSS13
CTSL
CTSB
EM
DM
EM
DM
Trachea
Bronchus
Bronchiole
Intestine
Nose
Airway
b
Intestinal organoids
Intestinal tissue
Stem cells
TA cells
Goblet cells
Enterocytes
c
Expansion
Differentiation
d
OFLM4
PCNA
FABP1
FCGBP
Log expression level
Stem cells
TA cells
Goblet cells
Enterocytes
e
OFLM4
PCNA
FABP1
FCGBP
Log expression level
Stem cells
TA cells
Goblet cells
Enterocytes

Supplementary figure 1 Bulk and single cell RNA sequencing reveals intestinal expression of host proteases involved in viral entry

a) Heatmaps depicting expression of host genes (top) and proteases (bottom) in intestinal, nose and airway organoids. Nose organoids were cultured in expansion (EM) or differentiation (DM) medium. Colored bar represents Z-score of log2 transformed values.
b) t-SNE maps displaying a newly generated human organoid single cell RNA sequencing atlas (left), and a dataset reanalyzed from[32] (right). Colors indicate different cell types.
c) t-SNE maps displaying the human organoid single cell sequencing atlas. Color codes indicate cells derived from expansion or differentiation medium.
d) t-SNE maps and violin plots displaying expression of host factors in the human intestinal organoid cell atlas. Bars in t-SNE maps display color-coded normalized unique transcript expression (logarithmic scale).
e) t-SNE maps and violin plots displaying expression of host factors in the human intestinal tissue atlas. Bars in t-SNE maps display color-coded normalized unique transcript expression (logarithmic scale).

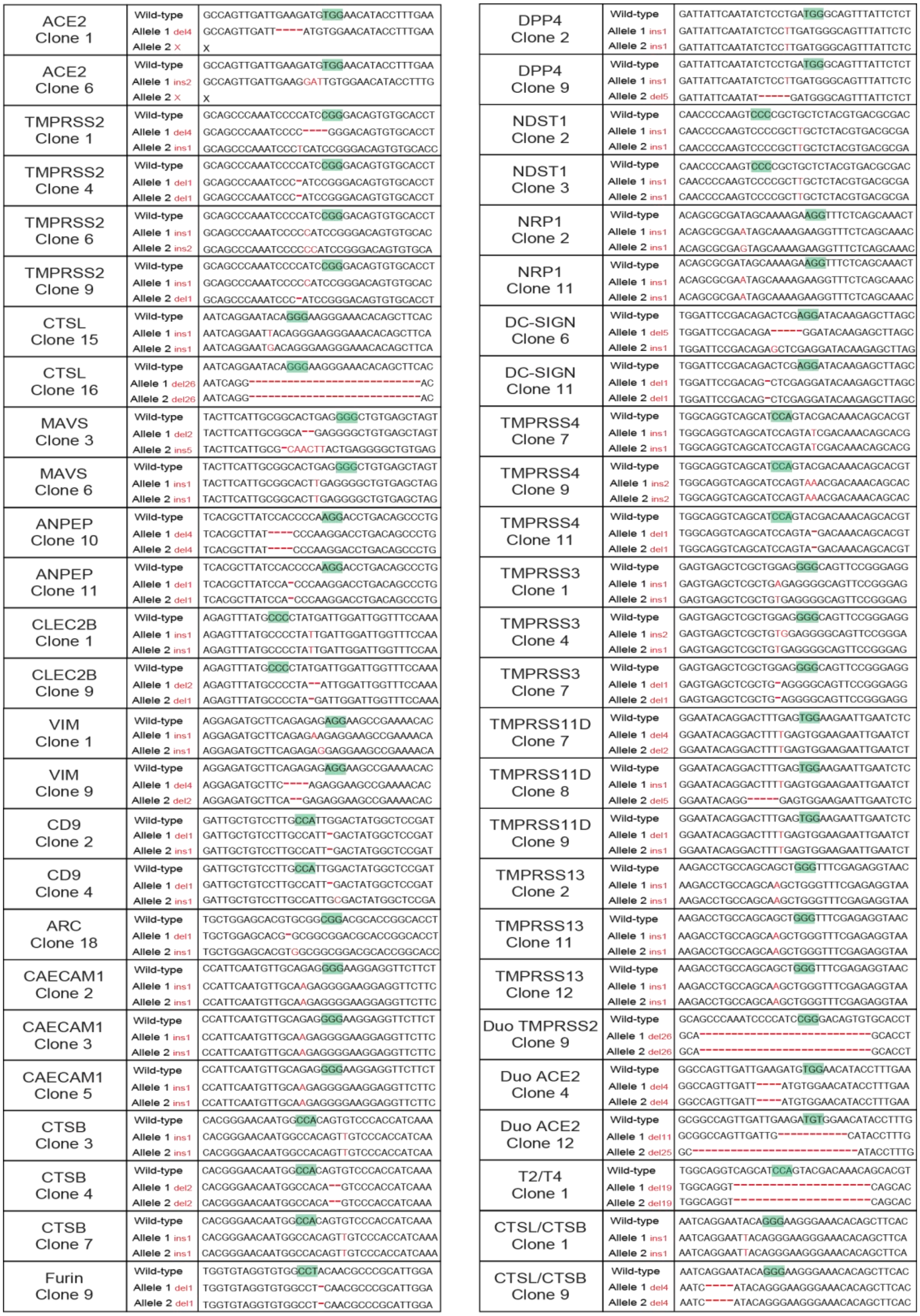

Supplementary figure 2 Generation of a host factor loss-of-function organoid biobank

Overview of the genetic alterations causing frameshifts in the different host factors. Green boxes indicate PAM sequence, red dashes or bases indicate respectively deletions and insertions.

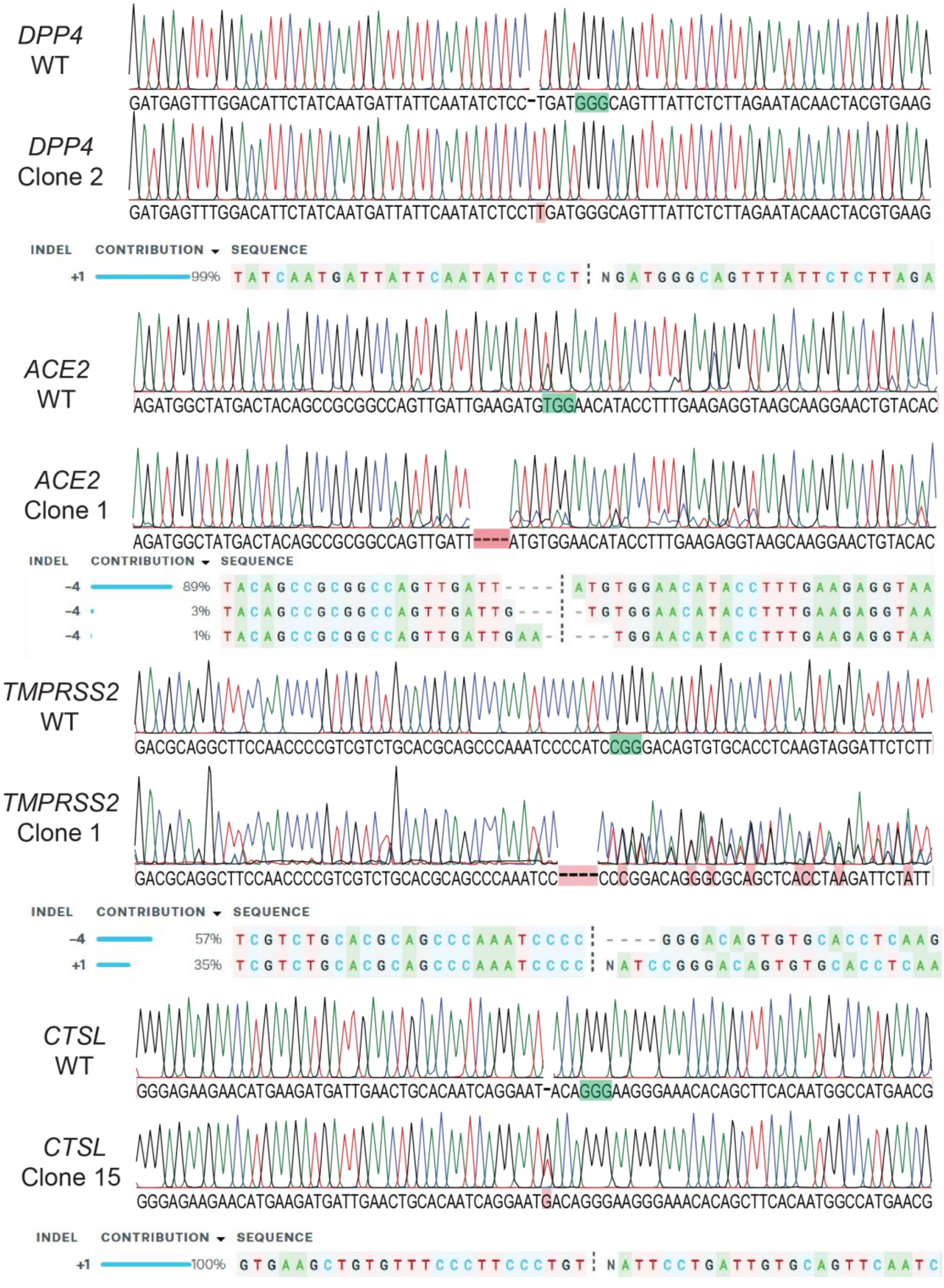

Supplementary figure 3 Organoid genotyping by sanger sequencing and *in silico* ICE analysis.

Sanger traces and subsequent *in silico* sanger deconvolution by ICE v2 for the first clone of ACE2, TMPRS2, DDP4 and CTSL indicating out-of-frame indel induction at the target site. Green boxes indicate PAM sequence.

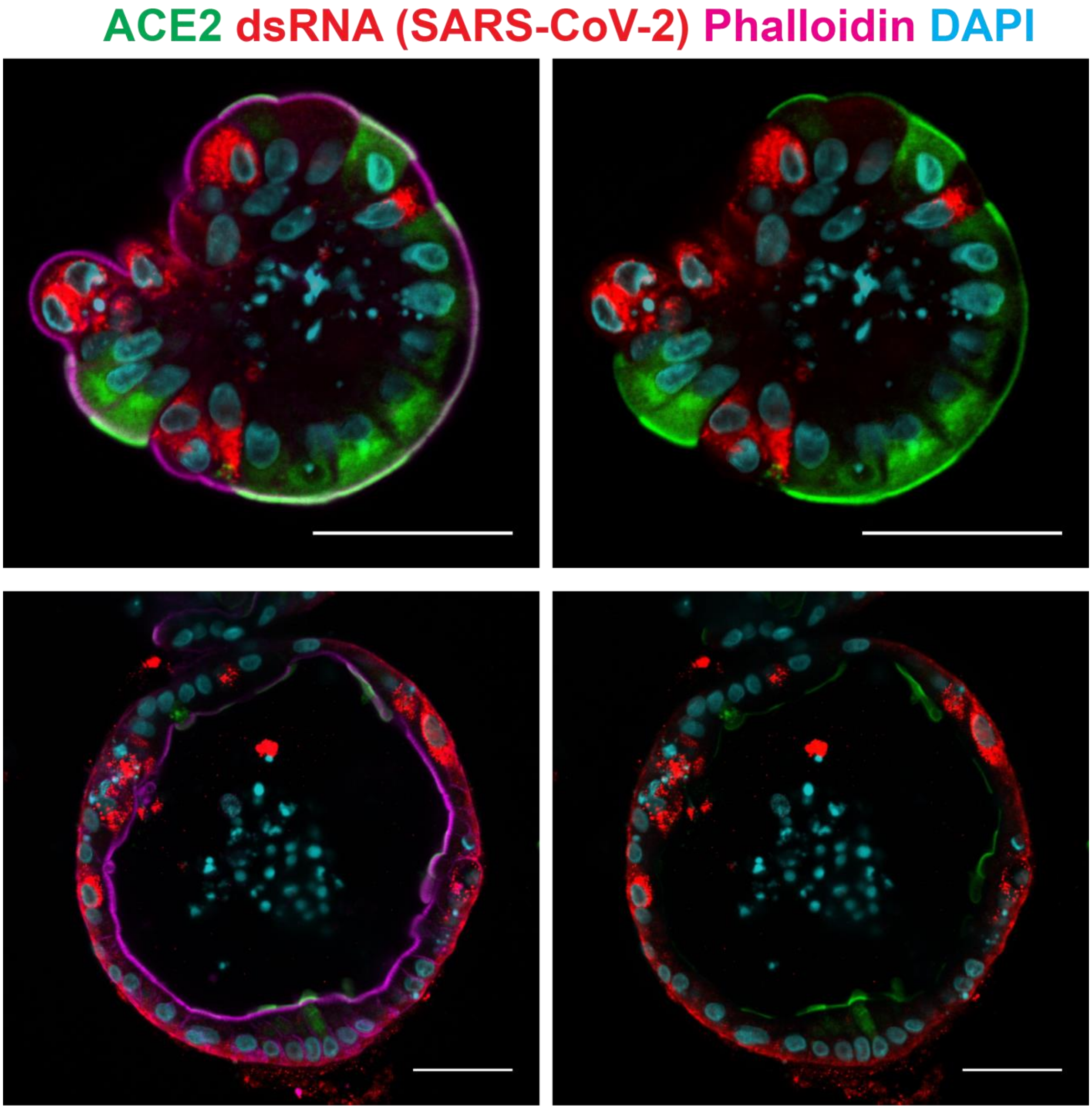

Supplementary figure 4 SARS-CoV-2 infected cells contain varying degrees of membranous ACE2 protein

Immunofluorescent staining of SARS-CoV-2 infected organoids. Virus is visualized by dsRNA. Some infected cells are devoid of visible ACE2 on the outer membrane. Scale bars are 50 µm. Experiment was repeated 2 times with similar results.

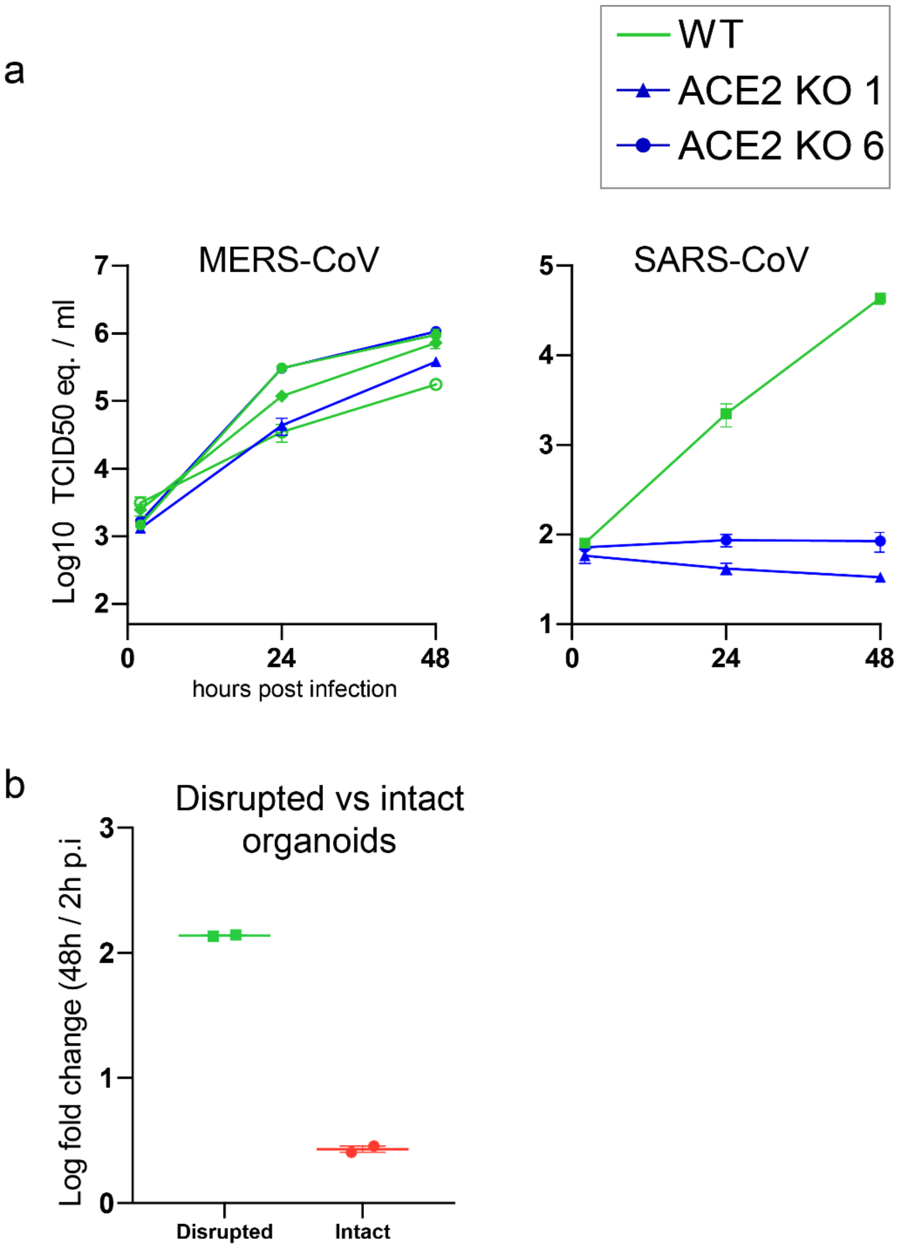

Supplementary figure 5 SARS-CoV but not MERS-CoV replication depends on ACE2

a) qPCR analysis targeting the N gene (SARS-CoV) or upE region (MERS-CoV) to quantify viral replication in WT and ACE2 KO organoids. Error bars represent SEM. Each data point represents the mean of 3 replicates.

b) qPCR analysis targeting the E gene to quantify viral replication of the SARS-CoV-2 in mechanically disrupted or intact organoids, where virus can only access the basolateral side. Graphs display the ratio between viral titer at 48 hours compared to 2 hours post infection (p.i.). Error bars represent SEM. Each data point represents the mean of 3 replicates.

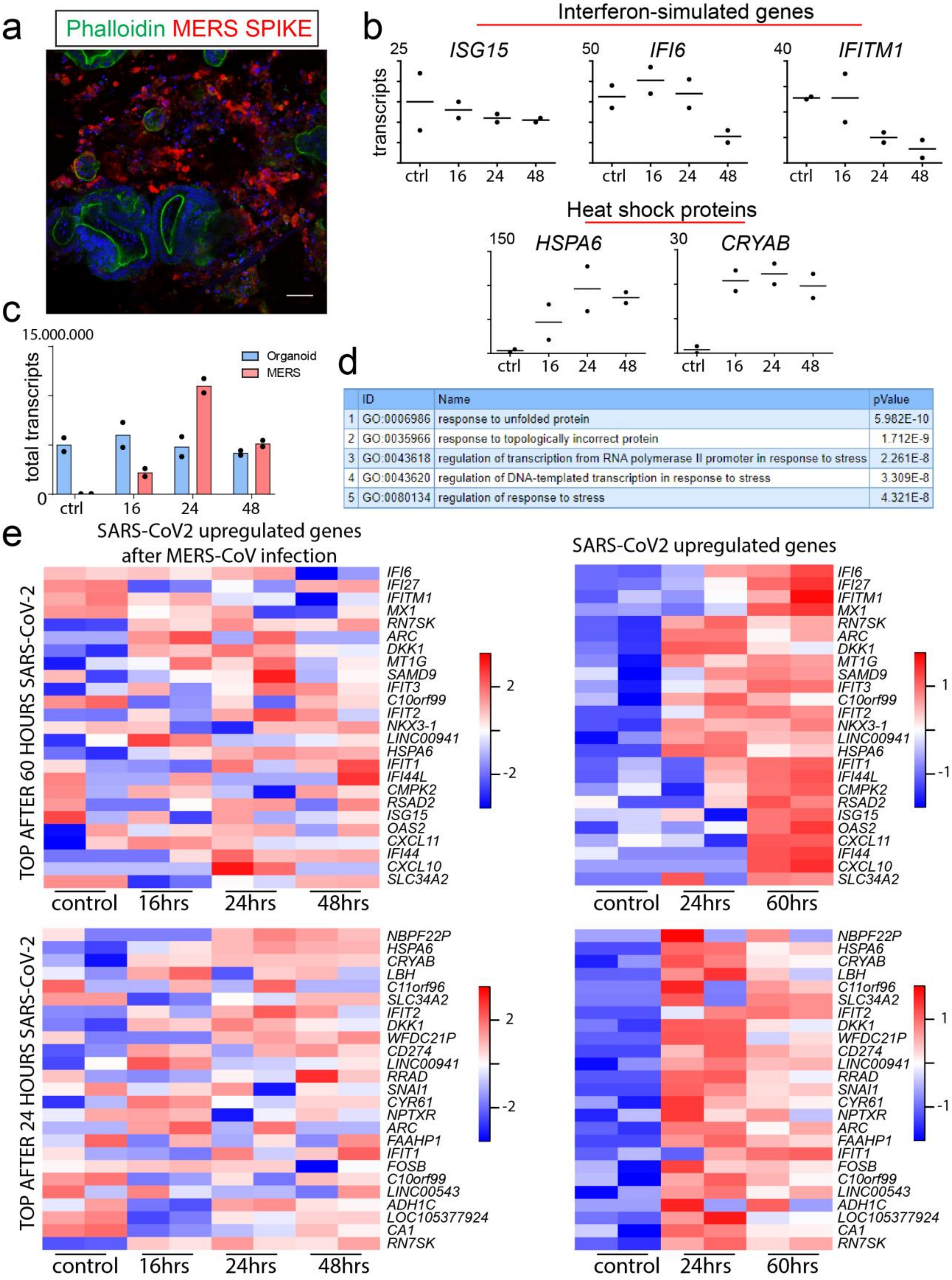

	ID	Name	pValue
1	GO:0006986	response to unfolded protein	5.982E-10
2	GO:0035966	response to topologically incorrect protein	1.712E-9
3	GO:0043618	regulation of transcription from RNA polymerase II promoter in response to stress	2.261E-8
4	GO:0043620	regulation of DNA-templated transcription in response to stress	3.309E-8
5	GO:0080134	regulation of response to stress	4.321E-8

Supplementary figure 6 Establishment of MERS infection model in human intestinal organoids

a) Immunofluorescent staining of organoids 48 hours after MERS-CoV infection. Virus is visualized by staining for the spike protein. The majority of the infected organoids display massive cell death. Scale bars are 50 µm. Experiment was repeated 3 times with similar results.

b) Graphs depicting the transcript counts determined by RNA sequencing of different genes upon MERS-CoV infection. Different numbers indicate timepoints (hours) after infection. n=2 technically independent samples.

c) Graph depicting the transcript counts mapping to human and MERS genomes in MERS-CoV infected organoids. MERS reads increase over time, but drop again at 48 hours potentially due to cell death of infected cells. For all other analyses, MERS reads were removed from analyses for normalization purposes. n=2 technically independent samples.

d) Go term enrichment analysis for biological processes of the 60 most significantly upregulated genes upon MERS-CoV infection in organoids. p-values are determined by random sampling from the whole genome. Bonferroni correction was performed to account for multiple comparisons.

e) Heatmap depicting the expression profile of the 25 genes with strongest upregulation upon SARS-CoV-2 infection[17]; right heatmaps) and the same genes upon MERS-CoV infection (left heatmaps). The top heatmaps show the most prominently upregulated genes after 60 hours of SARS-CoV-2 infection, the lower heatmaps after 24 hours. In contrast to SARS-CoV-2, MERS-CoV does not induce expression of ISGs. Colored bar represent Z-score of log2 transformed values.

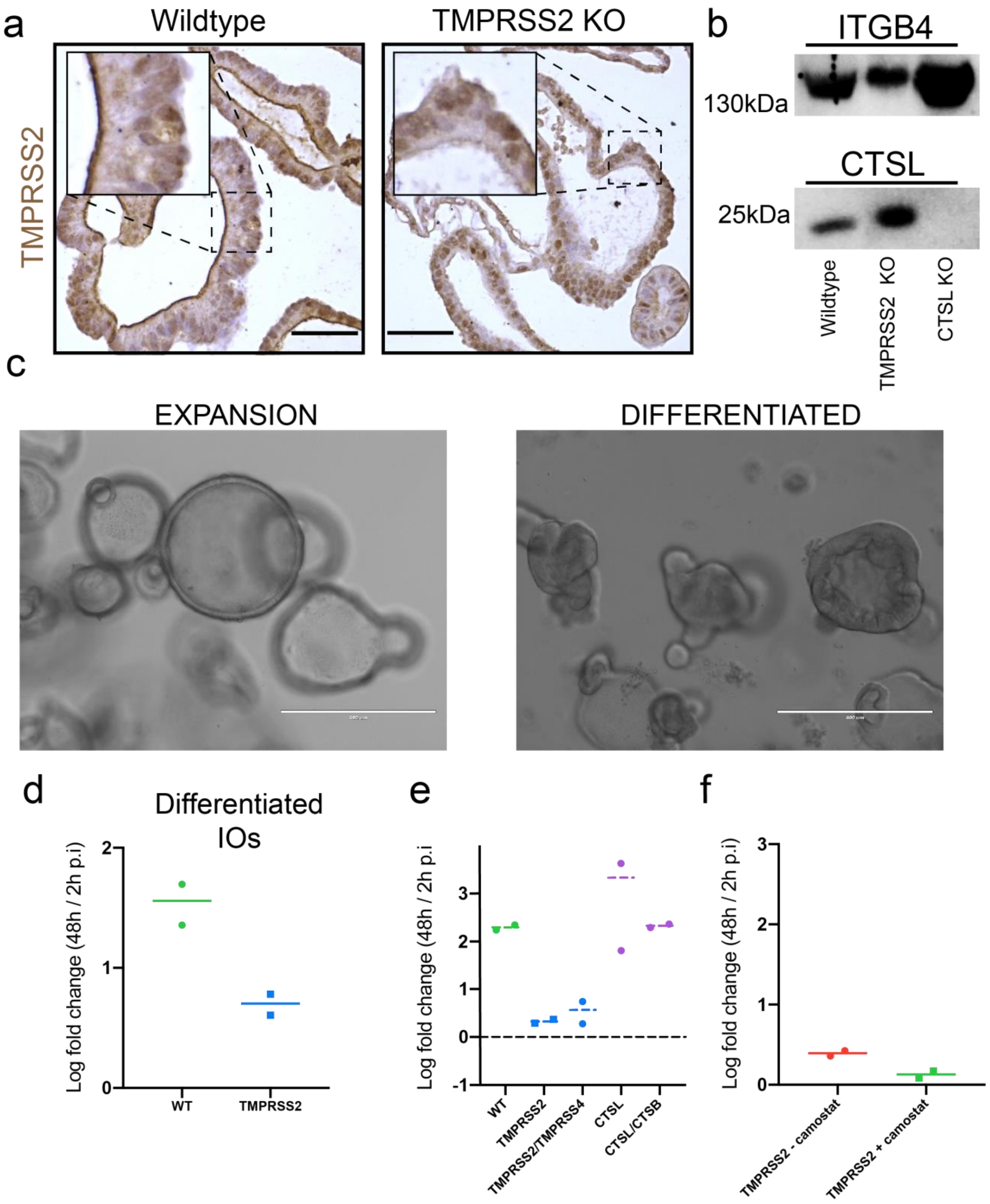

a
Wildtype
TMPRSS2 KO
TMPRSS2
b
ITGB4
130kDa
CTSL
25kDa
Wildtype
TMPRSS2 KO
CTSL KO
c
EXPANSION
DIFFERENTIATED
d
Differentiated IOs
Log fold change (48h / 2h p.i)
0
1
2
WT
TMPRSS2
e
Log fold change (48h / 2h p.i)
-1
0
1
2
3
WT
TMPRSS2
TMPRSS2/TMPRSS4
CTSL
CTSL/CTSB
f
Log fold change (48h / 2h p.i)
0
1
2
3
TMPRSS2 - camostat
TMPRSS2 + camostat

Supplementary figure 7 Lack of redundancy in cathepsins and serine proteases in viral entry

a) Immunohistochemical staining of TMPRSS2 in wildtype and TMPRSS2-knock out (KO) organoids. TMPRSS2 locates mostly to the apical membrane in wildtype cells, and is absent in mutant organoids. Scale bars are 50 µm.

b) Western blotting for CTSL and Integrin B4 (ITGB4, loading control, tested on the same gel) in wildtype, TMPRSS2- and CTSL-KO organoids. CTSL protein is completely lost in corresponding mutant organoids. Experiment was performed once.

c) Brightfield images of expanding and 5-day differentiated organoids that were infected with SARS-CoV-2. Scale bars are 400 µm. Experiment was repeated 6 times with similar results.

d) Graph displaying the ratio between the viral titer at 48 hours compared to 2 hours post infection (p.i.) quantified by qPCR targeting the E gene to measure viral replication of SARS-CoV-2 in expanding and 5-day differentiated intestinal organoid cells harboring a loss-of-function mutation in the TMPRSS2 gene. n=2 technically independent samples. Mean is indicated by colored bar.

e) qPCR analysis targeting the E gene to quantify viral replication of the SARS-CoV-2 in organoids harboring different single and double mutants in host proteases. Graphs display the ratio between viral titer at 48 hours compared to 2 hours post infection (p.i.). The dotted line indicates a fold change of 1. n=2 technically independent samples. Mean is indicated by colored and dotted bar.

f) qPCR analysis targeting the E gene to quantify viral replication of the SARS-CoV-2 in TMPRSS2-deficient organoids treated with the broad serine protease inhibitor Camostat. Graphs display the ratio between viral titer at 48 hours compared to 2 hours post infection (p.i.). n=2 technically independent samples. Mean is indicated by colored bar.

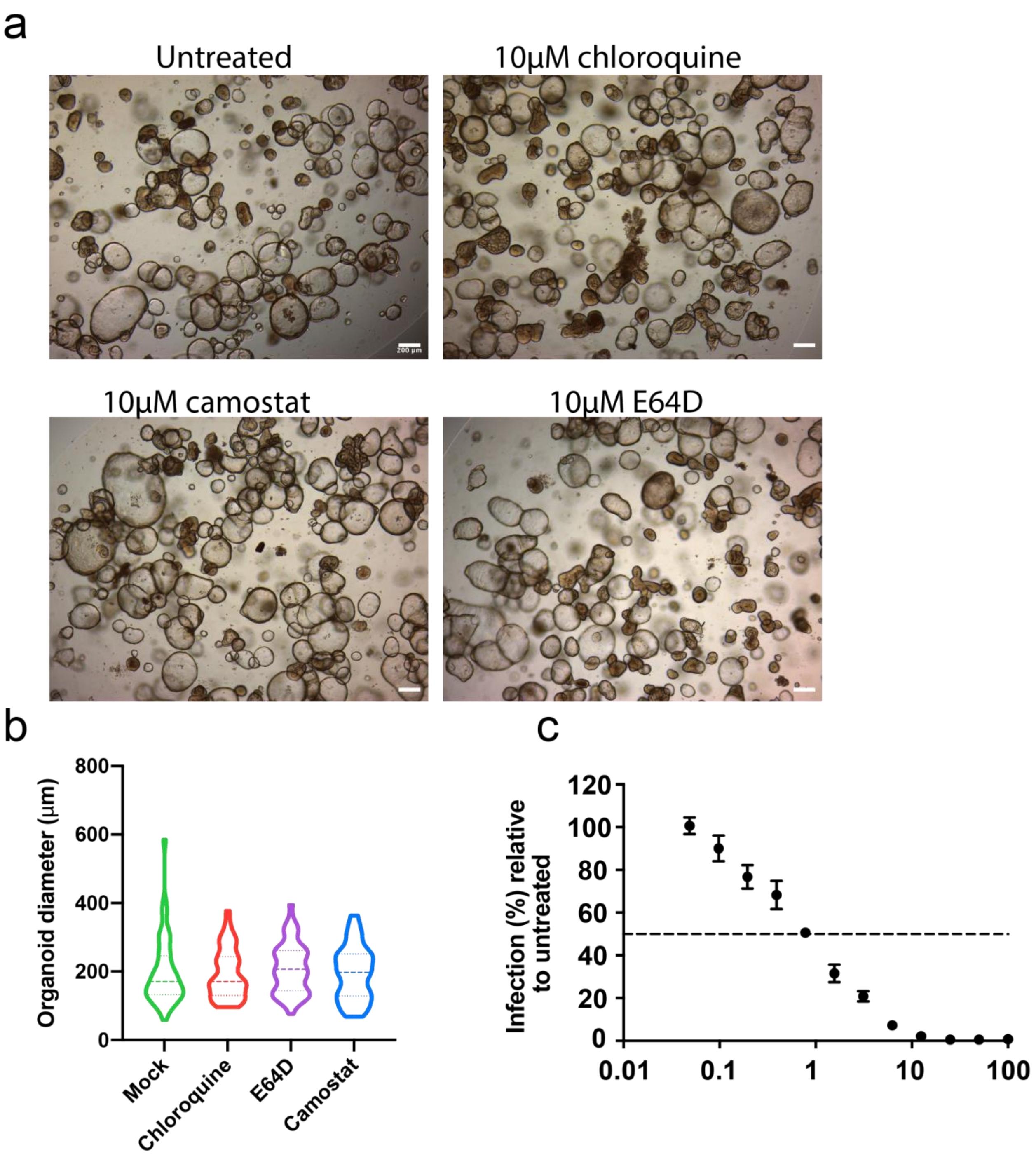
a
Untreated
10µM chloroquine
10µM camostat
10µM E64D
b
Organoid diameter (µm)
800
600
400
200
0
Mock
Chloroquine
E64D
Camostat
c
Infection (%) relative to untreated
120
100
80
60
40
20
0
0.01
0.1
1
10
100

Supplementary figure 8 Inhibition of serine proteases but not chloroquine inhibits viral replication in organoids

a) Brightfield images of organoids treated for 48 hours with serine protease inhibitor Camostat, chloroquine or cysteine protease inhibitor E64D. Scale bars are 200 µm.

b) Violin plot of average sizes in organoids from Fig. 6E. The diameter was measured in at least n=50 organoids per treatment. Organoid size was not significantly changed in any of the treatments, indicating similar growth. Dotted lines within the violins indicate the median and quartiles. Statistics were performed by one-way ANOVA followed by a multiple-comparison test (Original FDR method of Benjamini and Hochberg; Q = 0.05) on log10 transformed values. Exact p-values can be found in Supplementary dataset 5.

c) Quantification of viral entry in Vero E6 cells upon treatment with chloroquine using immunostaining 8 hours after infection. Error bars represent standard error of mean. N=3.

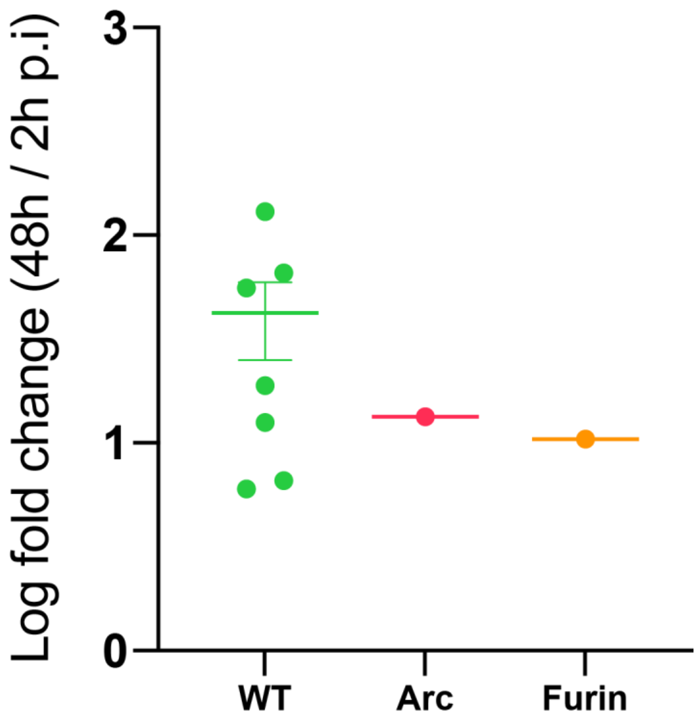

Supplementary figure 9 SARS-CoV-2 replication in Furin- en ARC-mutant IOs

Graph displaying the ratio between the viral titer at 48 hours compared to 2 hours post infection (p.i.) quantified by qPCR targeting the E gene to measure viral replication of SARS-CoV-2 in wildtype, and ARC- and Furin- mutant organoids. Experiment was performed with n=1 biological replicate. The WT data is displayed from Fig. 4A. Error bar represents standard error of mean.

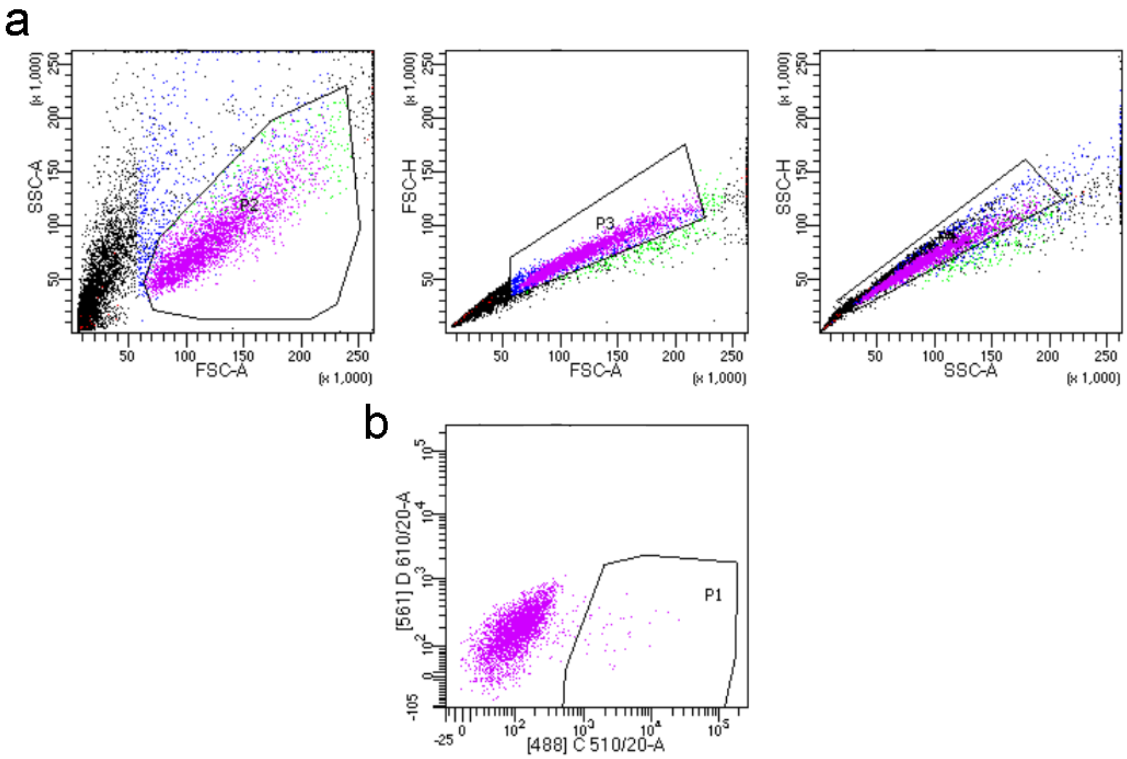

Supplementary figure 10 FACS gating strategy to select for transfected GFP positive cells

a) Three step gating strategy to select for single cells by using forward and side scatter.

b) To select for transfected cells, GFP positive cells that appear in gate P1 were sorted.

Gene	Guide	Exon	Forward cloning primer	Reverse cloning primer	Forward sequencing primer	Reverse sequencing primer
ACE2	CGGCCAGTTGATTGAAGATG	6	**CACCG**CGGCCAGTTGATTGAAGATG	**AAAC**CATCTTCAATCAACTGGCCG**C**	TTACCAGGGGTGAGGTGGAGCG	TGAGGCCTGGGAATGGCTGACC
DPP4	ATTATTCAATATCTCCTGAT	5	**CACCG**ATTATTCAATATCTCCTGAT	**AAAC**ATCAGGAGATATTGAATAAT**C**	CGCCACCTACCTTGCATCACGG	TCCTGCTTCAGCCTTCCCTCCT
TMPRSS2	ACGCAGCCCAAATCCCCATC	3	**CACCG**ACGCAGCCCAAATCCCCATC	**AAAC**GATGGGGATTTGGGCTGCGT**C**	ACCGGAAAACCCCTATCCCGCA	CGCAGGACCCTGAAGCCCAATG
CTSL	ACTGCACAATCAGGAATACA	3	**CACCG**ACTGCACAATCAGGAATACA	**AAAC**TGTATTCCTGATTGTGCAGT**C**	GCATCCCCAGAGGTGTCAAGCC	CCACAGATCTGGGGGCCTCATA
MAVS	TACTTCATTGCGGCACTGAG	3	**CACCG**TACTTCATTGCGGCACTGAG	**AAAC**CTCAGTGCCGCAATGAAGTA**C**	CTGGCTTGAGCAGGACAGTGGC	CGCATCGTCCCGCAGTATCACC
ANPEP	CATCACGCTTATCCACCCCA	3	**CACCG**CATCACGCTTATCCACCCCA	**AAAC**TGGGGTGGATAAGCGTGATG**C**	GTGGGAGCAGGAGCAGGGGTTA	GGGCGTGGTGTGGAACTCAGTG
VIM	GGAGGAGATGCTTCAGAGAG	3	**CACCG**GGAGGAGATGCTTCAGAGAG	**AAAC**CTCTCTGAAGCATCTCCTCC**C**	AATCACCGGGCGGGAGAAGGAA	CGCCACTAGAGGGCTTTACGCG
CD9	GAATCGGAGCCATAGTCCAA	3	**CACCG**GAATCGGAGCCATAGTCCAA	**AAAC**TTGGACTATGGCTCCGATTC**C**	AATGGGCGGCAGGTAGGTCACT	TGGGTCAACATGGGGCCTCCAA
ARC	AGATGCTGGAGCACGTGCGG	1	**CACCG**AGATGCTGGAGCACGTGCGG	**AAAC**CCGCACGTGCTCCAGCATCT**C**	CTGCGCACAGATGGAGCTGGAC	TCGTGGCAGTAGCTCTCGGGAC
CAECAM1	TGCCATTCAATGTTGCAGAG	2	**CACCG**TGCCATTCAATGTTGCAGAG	**AAAC**CTCTGCAACATTGAATGGCA**C**	GCTCAGGACCCAAGGCCCCATT	GGCCAGACCCTGACTGAGTCCT
CLEC2B	GGAAACCAATCCAATCATAG	3	**CACCG**GGAAACCAATCCAATCATAG	**AAAC**CTATGATTGGATTGGTTTCC**C**	ACCTGTGTCCAGTGATCTCAAA	CTGCCACGGGACCTCAGAGACA
FURIN	ACCTCCAATGCGGGCGTTGT	7	**CACCG**ACCTCCAATGCGGGCGTTGT	**AAAC**ACAACGCCCGCATTGGAGGT**C**	GCTTTTCCCACAGTCCTGGCCC	GCTGCATGGGTCCAGCTGTCAG
CEACAM5	GATGATGTTCTGGATCAGCA	2	**CACCG**GATGATGTTCTGGATCAGCA	**AAAC**TGCTGATCCAGAACATCATC**C**	GCTCAGGACCCAGGACCCCATT	GAACAAGTGTGGCCAGAAAGTCC
HSP5A	CGTGTTCAAGAACGGCCGCG	2	**CACCG**CGTGTTCAAGAACGGCCGCG	**AAAC**CGCGGCCGTTCTTGAACACG**C**	CTCCTGGTAAGTGGGGTTGCGG	TCTCGGGGTTGGAGGTGAGCTG
CD209	CAAGCTGCAGGAGATCTACC	4	**CACCG**CAAGCTGCAGGAGATCTACC	**AAAC**GGTAGATCTCCTGCAGCTTG**C**	CTCCTTGGCCTTCTGTGCTGCC	GCTCACCCACTGCAGCCTTCAG
TMPRSS4	AGACGTGCTGTTTGTCGTAC	8	**CACCG**AGACGTGCTGTTTGTCGTAC	**AAAC**GTACGACAAACAGCACGTCT**C**	TCTCTCCACTCTGGCCCCA	GCCCTTCCCTGAGGTAAGGCCA
NDST1	TCGCGTCACGTAGAGCAGCG	3	**CACCG**TCGCGTCACGTAGAGCAGCG	**AAAC**CGCTGCTCTACGTGACGCGA**C**	GGTGAGCCCTCCATGAATGT	GTGTGGGATGGACTCAGACG
**Nucleotides depicted in bold show primer overhangs needed for sgRNA cloning into PX458**						

Supplementary table 1 Oligos used in this study as gRNAs and sequencing primers
