# Supplementary material for: A CRISPR/Cas9 genetically engineered organoid biobank reveals essential host factors for coronaviruses
Source: Nat Commun. 2021 Sep 17;12:5498. doi: 10.1038/s41467-021-25729-7 (PMC8448725; doi:10.1038/s41467-021-25729-7)
Supplement: Supplementary file 2 — Description of Additional Supplementary Files [file 41467_2021_25729_MOESM2_ESM.docx]

**Description of Additional Supplementary Files**

**Title: Supplementary dataset 1 Normalized transcript counts in intestinal and airway organoids**

**Description:**Table shows normalized transcript counts determined by RNA sequencing of duplicate organoids from the respective regions, and intestinal organoids infected with SARS-CoV-2. The intestinal organoid dataset was obtained from^17^.

**Title: Supplementary dataset 2**

**Description:** Overview of genetically modified organoids generated in this study

**Title: Supplementary dataset 3 Normalized transcript counts in MERS-CoV infected organoids**

**Description:** Table shows normalized transcript counts determined by RNA sequencing of a duplicate control treatment (NC), and 16, 24 and 48 hours after MERS-CoV infection.

**Title: Supplementary dataset 4 Differentially regulated genes upon MERS-CoV infection in organoids**

**Description:** Fold change in gene expression versus control after 48 hours of MERS-CoV infection. p-values were determined using a two-tailed Wald test. Benjamini-Hochberg correction is performed to account for multiple hypothesis testing.
