# Supplementary material for: A CRISPR/Cas9 genetically engineered organoid biobank reveals essential host factors for coronaviruses
Source: Nat Commun. 2021 Sep 17;12:5498. doi: 10.1038/s41467-021-25729-7 (PMC8448725; doi:10.1038/s41467-021-25729-7)
Supplement: Supplementary file 8 — Reporting Summary [file 41467_2021_25729_MOESM8_ESM.pdf]

nature research

Corresponding author(s): Bart Haagmans, Hans Clevers

Last updated by author(s): Aug 9, 2021

Reporting Summary

Nature Research wishes to improve the reproducibility of the work that we publish. This form provides structure for consistency and transparency in reporting. For further information on Nature Research policies, see our Editorial Policies and the Editorial Policy Checklist.

Statistics

For all statistical analyses, confirm that the following items are present in the figure legend, table legend, main text, or Methods section.

n/a	Confirmed	
☐	☒	The exact sample size (n) for each experimental group/condition, given as a discrete number and unit of measurement
☒	☐	A statement on whether measurements were taken from distinct samples or whether the same sample was measured repeatedly
☐	☒	The statistical test(s) used AND whether they are one- or two-sided<br>*Only common tests should be described solely by name; describe more complex techniques in the Methods section.*
☒	☐	A description of all covariates tested
☒	☐	A description of any assumptions or corrections, such as tests of normality and adjustment for multiple comparisons
☐	☒	A full description of the statistical parameters including central tendency (e.g. means) or other basic estimates (e.g. regression coefficient) AND variation (e.g. standard deviation) or associated estimates of uncertainty (e.g. confidence intervals)
☐	☒	For null hypothesis testing, the test statistic (e.g. F, t, r) with confidence intervals, effect sizes, degrees of freedom and P value noted<br>*Give P values as exact values whenever suitable.*
☒	☐	For Bayesian analysis, information on the choice of priors and Markov chain Monte Carlo settings
☒	☐	For hierarchical and complex designs, identification of the appropriate level for tests and full reporting of outcomes
☒	☐	Estimates of effect sizes (e.g. Cohen's d, Pearson's r), indicating how they were calculated

Our web collection on statistics for biologists contains articles on many of the points above.

Software and code

Policy information about availability of computer code

Data collection	Leica LAS X Version 1.1 (imaging), Bio-Rad CFX Manager Version 3.1. (quantitative PCR)
Data analysis	RNA sequencing data was analyzed using available scripts (DeSEQ2 (v1.20), RaceID3, stated in the methods section) using R Studio software. Software versions used in this manuscript: ImageJ (Fiji, Version 1.51n), R Studio (1.1.453), Leica LAS X Version 1.1 and Graphpad Prism 8.2.1

For manuscripts utilizing custom algorithms or software that are central to the research but not yet described in published literature, software must be made available to editors and reviewers. We strongly encourage code deposition in a community repository (e.g. GitHub). See the Nature Research guidelines for submitting code & software for further information.

Data

Policy information about availability of data

All manuscripts must include a data availability statement. This statement should provide the following information, where applicable:

- Accession codes, unique identifiers, or web links for publicly available datasets
- A list of figures that have associated raw data
- A description of any restrictions on data availability

Bulk and single-cell RNA–seq data that support the findings of this study have been deposited in the Gene Expression Omnibus (GEO).

Field-specific reporting

Please select the one below that is the best fit for your research. If you are not sure, read the appropriate sections before making your selection.

[x] Life sciences [ ] Behavioural & social sciences [ ] Ecological, evolutionary & environmental sciences

For a reference copy of the document with all sections, see nature.com/documents/nr-reporting-summary-flat.pdf

Life sciences study design

All studies must disclose on these points even when the disclosure is negative.

Sample size	No sample-size calculation was performed. We decided to generate and perform all experiments on at least 2 mutant organoid lines per gene that in our experience is sufficient to prevent incorrect conclusions based on CRISPR-CAS9 off-targets. A selected set of genes that significantly impacted viral replication were additionally mutated in 2 independent organoid lines from other patients to substantiate conclusions.
Data exclusions	No data were excluded
Replication	All attempts at replication were succesfull. Where relevant, every figure legend indicates how often experiments were replicated: Figure S7B has been performed once, other experiments were replicated 2-6 times.
Randomization	No samples were allocated into experimental groups in this work
Blinding	Single-cell and bulk mRNA-sequencing analysis was performed in an unbiased fashion with pooling all data, and was performed blinded during data collection. Analysis was performed blinded. Experimental conditions were assigned following initial analysis.<br><br>Viral replication measures were performed blinded.<br><br>All immunofluorescence and immunohistochemistry imaging were performed blinded: sample identities were allocated after data collection and anaylsis.

Reporting for specific materials, systems and methods

We require information from authors about some types of materials, experimental systems and methods used in many studies. Here, indicate whether each material, system or method listed is relevant to your study. If you are not sure if a list item applies to your research, read the appropriate section before selecting a response.

Materials & experimental systems

n/a	Involved in the study	
[ ]	[x]	Antibodies
[ ]	[x]	Eukaryotic cell lines
[x]	[ ]	Palaeontology and archaeology
[x]	[ ]	Animals and other organisms
[ ]	[x]	Human research participants
[x]	[ ]	Clinical data
[x]	[ ]	Dual use research of concern

Methods

n/a	Involved in the study	
[x]	[ ]	ChIP-seq
[x]	[ ]	Flow cytometry
[x]	[ ]	MRI-based neuroimaging

Antibodies

Antibodies used

Antibodies used in this study
A) mouse anti-nucleoprotein (1:200; 40143-MM05, Sino Biological), lot #HB14JL0605
B) mouse anti-dsRNA (1:200; #10010200, Scicons), lot #J2-2004
C) goat anti-ACE2 (1:100; AF933, R&D Systems), lot #HOK0320041
D) goat anti-DPP-4 (1:200; AF1180, R&D systems) lot # JOQ0218071
E) rabbit anti-MERS S1 (1:200; 40069-T52, Sino Biological) lot # HD09AP2707-B
F) rabbit anti-TMPRSS2 (1:100; ab109131, Abcam)
G) mouse anti-CTSL (1:100; # BMS1032, eBioscience)
H) anti-ITGB4 (1:100, Origene; AM33010PU-N)
I) donkey anti-mouse Alexa647 (1:1000, # A-31571, Thermofisher Scientific)
J) donkey anti-rabbit Alexa488 (1:1000, # A-21206, Thermofisher Scientific)
J) donkey anti-rabbit Alexa568 (1:1000, # A-10042, Thermofisher Scientific)
K) donkey anti-goat Alexa488 (1:1000, # A-11055, Thermofisher Scientific)

L) donkey anti-goat Alexa568 (1:1000, # A-11057, Thermofisher Scientific)
M) donkey anti-goat Alexa647 (1:1000, # A-21447,Thermofisher Scientific)
N) donkey anti-mouse Alexa568 (1:1000, # A-10037, Thermofisher Scientific)
O) Rabbit Anti-Mouse Immunoglobulins/HRP (1:2000, P0260, DAKO)
P) Goat Anti-Rabbit Immunoglobulins/HRP (1:2000, P0448, DAKO)

Validation

A) Immunofluorescence: Antibody has not been validated by the supplier for the used purpose. Immunostaining is consistent with expected pattern (and absent in non-coronavirus-infected, negative control).
B) Immunofluorescence: Antibody has been approved by the supplier for the used purpose.
C) Immunofluorescence: Antibody has been approved by the supplier for the used purpose.
D) Immunofluorescence: Antibody has been approved by the supplier for the used purpose.
E) Immunofluorescence: Antibody has not been validated by the supplier for the used purpose. Immunostaining is consistent with expected pattern (and absent in non-MERS-infected, negative control).
F) Immunohistochemistry-paraffin: Antibody has been approved by the supplier for the used purpose.
G) Western blotting: Antibody has been approved by the supplier for the used purpose.
H) Western blotting: Antibody has been approved by the supplier for the used purpose.
I) Immunofluorescence: Antibody has been approved by the supplier for the used purpose.
J) Immunofluorescence: Antibody has been approved by the supplier for the used purpose.
K) Immunofluorescence: Antibody has been approved by the supplier for the used purpose.
L) Immunofluorescence: Antibody has been approved by the supplier for the used purpose.
M) Immunofluorescence: Antibody has been approved by the supplier for the used purpose.
N) Immunofluorescence: Antibody has been approved by the supplier for the used purpose.
O) Western blotting: Antibody has been approved by the supplier for the used purpose.
P) Immunohistochemistry-parafin: Antibody has been approved by the supplier for the used purpose.

Eukaryotic cell lines

Policy information about cell lines

Cell line source(s)

Vero and VeroE6 cells were obtained from ATCC.

Authentication

No further authentication procedures were done.

Mycoplasma contamination

All cell lines tested negative for mycoplasma.

Commonly misidentified lines
(See ICLAC register)

N/A

Human research participants

Policy information about studies involving human research participants

Population characteristics

Adult intestinal tissue: The patients were operated for a colorectal tumor, and a sample from non-transformed, normal mucosa was taken for this study.

Adult lung tissue : Adult lung tissue was obtained from residual, tumor-free, material obtained at lung resection surgery for lung cancer.

Nasal inferior turbinate brushes: Nasal inferior turbinate brushes were obtained from the Hadassah Medical Center, Jerusalem, with informed consent of the patient. Patients were diagnosed with primary ciliary dyskinesia, and tissue was obtained from healthy donors as a comparison. Healthy material was used for the RNA sequencing in this work.

Recruitment

Patients were randomly recruited from a group with above diagnosis.

Ethics oversight

Adult intestinal tissue: The study was approved by the UMC Utrecht (Utrecht, The Netherlands) ethical committee and was in accordance with the Declaration of Helsinki and according to Dutch law. This study is compliant with all relevant ethical regulations regarding research involving human participants.

Adult lung tissue: The Medical Ethical Committee of the Erasmus MC Rotterdam granted permission for obtaining the adult lung tissue for study (METC 2012-512).

Nasal inferior turbinate brushes: The study was approved by the ethical committee (Hadassah Medical Organization (HMO) IRB committee) and was in accordance with the Declaration of Helsinki and according to Israeli law under IRB approval number 075-16 HMO.

Note that full information on the approval of the study protocol must also be provided in the manuscript.
